# Investigating the effectiveness of electronically delivered cognitive behavioural therapy (e-CBTi) compared to pharmaceutical interventions in treating insomnia: Protocol for a randomized controlled trial

**DOI:** 10.1371/journal.pone.0285757

**Published:** 2023-05-16

**Authors:** Yiran Zhu, Callum Stephenson, Elnaz Moghimi, Jasleen Jagayat, Niloofar Nikjoo, Anchan Kumar, Amirhossein Shirazi, Charmy Patel, Mohsen Omrani, Nazanin Alavi

**Affiliations:** 1 Faculty of Health Sciences, Queen’s University, Kingston, Ontario, Canada; 2 Faculty of Health Sciences, Department of Psychiatry, Queen’s University, Kingston, Ontario, Canada; 3 Faculty of Health Sciences, Centre for Neuroscience Studies, Queen’s University, Kingston, Ontario, Canada; 4 OPTT Inc., Toronto, Ontario, Canada; University Hospital Cologne: Uniklinik Koln, GERMANY

## Abstract

**Background:**

Insomnia is one of the most prevalent sleep disorders characterized by an inability to fall or stay asleep. Available treatments include pharmacotherapy and cognitive behavioural therapy for insomnia (CBTi). Although CBTi is the first-line treatment, it has limited availability. Therapist-guided electronic delivery of CBT for insomnia (e-CBTi) offers scalable solutions to enhance access to CBTi. While e-CBTi produces comparable outcomes to in-person CBTi, there is a lack of comparison to active pharmacotherapies. Therefore, direct comparisons between e-CBTi and trazodone, one of the most frequently prescribed medications for insomnia, is essential in establishing the effectiveness of this novel digital therapy in the health care system.

**Objective:**

The aim of this study is to compare the effectiveness of a therapist-guided electronically-delivered cognitive behavioural therapy (e-CBTi) program to trazodone in patients with insomnia.

**Methods:**

Patients (n = 60) will be randomly assigned to two groups: treatment as usual (TAU) + trazodone and TAU + e-CBTi for seven weeks. Each weekly sleep module will be delivered through the Online Psychotherapy Tool (OPTT), a secure, online mental health care delivery platform. Changes in insomnia symptoms will be evaluated throughout the study using clinically validated symptomatology questionnaires, Fitbits, and other behavioural variables.

**Results:**

Participant recruitment began in November 2021. To date, 18 participants have been recruited. Data collection is expected to conclude by December 2022 and analyses are expected to be completed by January 2023.

**Conclusions:**

This comparative study will improve our understanding of the efficacy of therapist-guided e-CBTi in managing insomnia. These findings can be used to develop more accessible and effective treatment options and influence clinical practices for insomnia to further expand mental health care capacity in this population.

**Trial registration:**

ClinicalTrials.gov (NCT05125146).

## Introduction

Insomnia is a common sleep disorder characterized by difficulties with sleep initiation, consolidation, duration, quality, and maintenance [1]. As a result, insomnia can have deleterious impacts on health, leading to increased risk of mental illnesses, accidents, poor physical health, and shortened life expectancy [1,2]. The disorder is prevalent in 10–30% of the population, particularly common in older adults, females, and individuals with physical and mental comorbidities [3].

Various biological, cognitive, and behavioural mechanisms that regulate sleep can contribute to insomnia. For example, the disorder is associated with physiological hyperarousal, including hypothalamic-pituitary-adrenal (HPA) axis hyperactivity [1,4]. Moreover, cognitive-emotional hyperarousal and sleep misperceptions may also contribute to insomnia [1,4]. Thus, pharmacotherapy that decreases physiologic arousal and cognitive behavioural therapy for insomnia (CBTi) that alters psychological arousal have been the most supported therapeutic approaches for managing insomnia [5,6].

Pharmacotherapy is frequently used to treat insomnia [6]. Benzodiazepine and nonbenzodiazepine drugs such as zopiclone are clinically effective in treating insomnia [5]. However, dependence, tolerance, abuse potential, and adverse effects (e.g., memory impairment, irritability, and confusion) are commonly reported side effects [7,8]. Since insomnia is associated with HPA axis activation, trazodone, the most commonly prescribed sleep aid, aims to counter this effect [7,9]. In a sample of 781 790 adults diagnosed with insomnia, 14.46% of adults used trazodone (95% CI, 14.38%-14.54%) [10]. This antidepressant is often prescribed at doses ranging between 25mg-100mg—lower than what is required for the treatment of depression to achieve sedative and hypnotic effects [7,9]. The prescribed doses reduce the occurrence of tolerance and drug dependency, providing clinical benefits with fewer side effects than benzodiazepine and nonbenzodiazepine drugs [9]. A systematic review of 45 studies provided adequate evidence supporting low-dose trazodone’s safety and efficacy in increasing sleep duration, improving patient-related sleep quality, and decreasing sleep latency when compared to a placebo [11]. However, medication non-adherence and patient preference to avoid pharmaceutical treatment are common challenges in treating psychiatric disorders, like insomnia [12,13]. Uptake of psychotropic medication can be limited due to treatment complexity, stigma, drug-drug interactions, lack of professional support, and medication costs [12]. Furthermore, evidence indicates that for the treatment of psychiatric disorders, patients yielded a significant three-fold preference for psychological treatment relative to pharmaceutical treatment [13]. As a result, the sustainability of pharmaceutical interventions for insomnia is questionable.

An alternative treatment approach is CBTi. Considered the first-line treatment for people with insomnia, CBT is typically delivered in-person, individually or in a group by a trained clinician [14]. CBTi aims to help patients recognize thoughts and behaviours associated with poor sleep and equip them with tools to change their maladaptive cognitive-behavioural patterns [15]. Core components of CBTi include cognitive therapy, stimulus control, sleep restriction, sleep hygiene, and relaxation training [15]. By encouraging patients to use skills and strategies to continually manage their sleep [16], CBT effectively improves insomnia severity, sleep quality, total sleep time, and other sleep-related parameters compared to waitlist control, placebo, or pharmaceutical interventions [17–19]. Additionally, there are fewer side effects, more durable sleep improvements, and fewer relapse episodes than most sleep medications, making it a more optimal treatment option for insomnia [15,20]. While CBTi is efficacious in many patients, several challenges remain in the dissemination of CBTi, including high costs, limited therapist availability, and inadequate accessibility to treatment [21]. Therefore, reducing barriers while maintaining high-quality care is imperative to improving the access and adherence to CBTi.

To address the limitations of in-person care, electronically delivered CBT (e-CBT) emerged as an alternative psychological approach to managing mental illnesses [22]. E-CBT typically consists of 8 to 12 patient-guided and goal-oriented pre-designed sessions [23] that are delivered via telephone, mobile applications, or the web [22]. Here, patients can access psychoeducational materials, practice CBT exercises, and complete worksheets relevant to their mental health challenges through therapist-guided or self-directed formats [22]. Therapist guidance can occur through synchronous (e.g., telephone, videoconferencing) or asynchronous (e.g., secured email, web-based platform communications) interactions. Moreover, e-CBT offers several advantages that can overcome the limitations of in-person CBT, including a low access threshold, reduced costs, and high scalability [24 Wilhelm]. Much of these advantages are a result of patient-therapist time being only a fraction of what is required by traditional in-person methods [24,25]. Since the content and delivery of e-CBT can be easily adapted for the condition being treated, it has shown significant progress in effectively managing various psychiatric disorders, including insomnia [26–28].

E-CBT for insomnia (E-CBTi) could play a key role in enhancing the dissemination of CBTi and improving the accessibility to care. Current literature has highlighted the superiority of e-CBTi to waitlist control, demonstrating significant improvements in total sleep time, sleep quality, and greater reductions in insomnia severity [28]. Furthermore, evidence has shown e-CBTi to have equivalent efficacy to in-person CBTi with no statistically significant differences between sleep efficiency, total sleep time and insomnia severity [29]. While e-CBTi can be an effective and more accessible treatment option for managing insomnia, its mode of delivery has mainly been through self-directed formats without therapist guidance or coaching [22,29]. Self-directed e-CBTi programs (e.g. Sleepio, SHUTi, NightOwl) can be beneficial [22]. However, therapist guidance via synchronous or asynchronous communications and personalized feedback in e-CBT may further improve patient adherence and promote desired treatment outcomes [22,30,31]. At the same time, synchronous therapy programs can be time-consuming, costly and may not be accessible to many patients. Asynchronous online communications, which do not rely on the simultaneous presence of therapists and patients, could be a viable alternative to lower therapy costs, increase care capacity, and improve adherence to e-CBTi [32]. For instance, the “I-Sleep’’ program that delivers e-CBTi along with asynchronous email interactions demonstrated more significant improvements in sleep quality and efficiency than waitlist control [33,34]. Since email communications are riddled with privacy concerns, potential confidentiality breaches, and lack of personalized care, a safe and secured clinic-like web-based interface such as the Online Psychotherapy Tool (OPTT; OPTT Inc.) might be a scalable solution [35–37]. Asynchronous communication with therapists through this platform may provide a safer and more cost-effective opportunity to increase engagement and adherence with e-CBTi.

To the best of our knowledge, studies that examine the effectiveness of asynchronous therapist-guided e-CBTi are limited. While it is known that e-CBTi produces better results compared to waitlist control groups and is comparable to in-person CBTi, its direct comparative effectiveness to active pharmaceutical interventions has not been established [29]. Trazodone is chosen as the representative for pharmacotherapy in this study because of its low abuse potential, high efficacy, and common usage in general practice [7,9,11]. Direct comparisons between trazodone and the scalable therapist-guided e-CBTi in clinical populations would be an important step in benchmarking the effectiveness and utility of this digital therapy in healthcare. The findings of this study will demonstrate the possibility of a novel alternative for treatment of insomnia. The suggested method could be more accessible, available and affordable than in-person CBTi while producing more significant long-term beneficial effects than trazodone in managing insomnia.

### Objective

The primary objective of the current study is to evaluate the comparative effectiveness of therapist-guided e-CBTi to the pharmaceutical drug, trazodone in managing insomnia. It is hypothesized that therapist-guided e-CBTi with personalized feedback will lead to more significant improvement in sleep outcomes when compared to trazodone. The treatment effect of therapist-guided e-CBTi and trazodone on insomnia severity and sleep will be evaluated using online questionnaires, sleep diaries, wearable sensors (Fitbit), and other behavioural variables.

## Materials and methods

### Study design and setting

**Fig 1:**
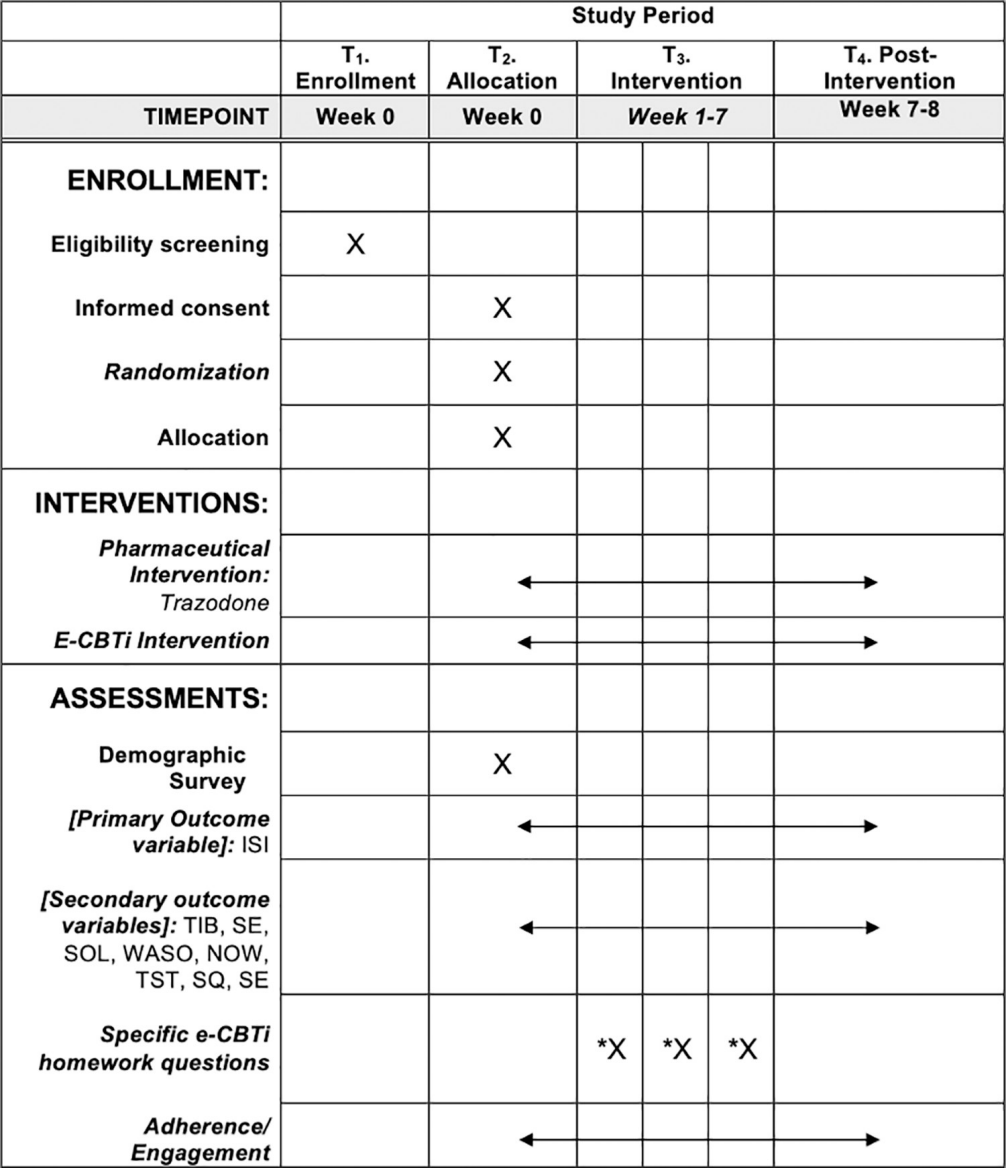
Standard Protocol Items: Recommendations for Interventional Trials (SPIRIT) schedule of enrollment, interventions, and assessments. Note: T = Timepoint; T1 and T2 = Baseline; T3 = Intervention; T4 = post 7-week intervention. ISI = Insomnia Severity Index; TIB = Time in Bed; SE = Sleep Efficiency; SOL = Sleep Onset Latency; WASO = Wake Time after Sleep Onset; NOW = Number of Nocturnal Awakenings; TST = Total Sleep Time; SQ = Sleep Quality. Adherence/Engagement = the number of logins; the number of homework and questionnaires completed; the number treatment sessions completed; compliance to keeping a consistent sleep schedule, avoiding naps, eliminating consuming alcohol or nicotine 2–3 hours before going to bed, getting out of bed shortly after waking and completing thought examination practices * = assessments for the e-CBTi group only.

**Fig 2:**
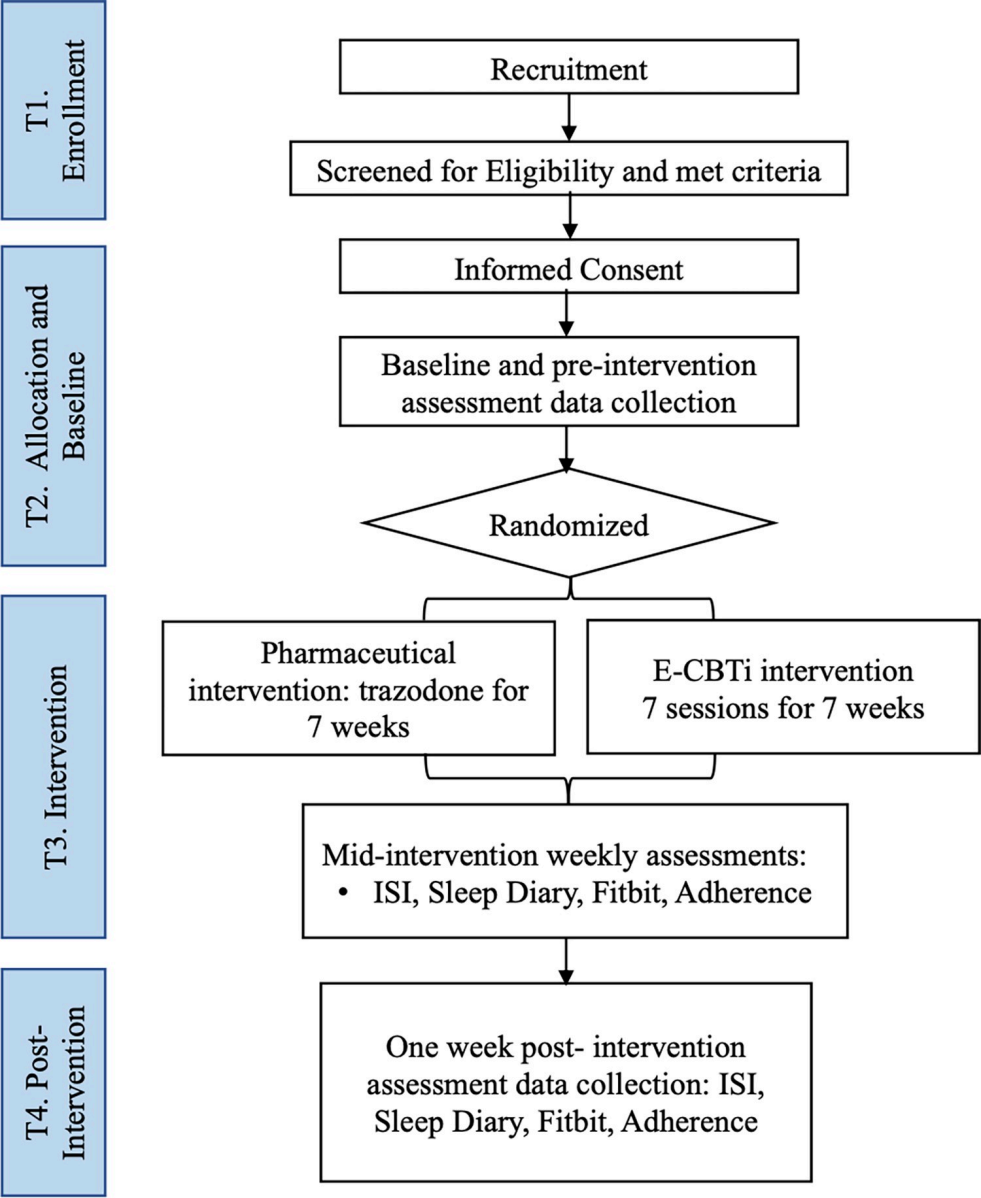
Participant flow chart.

This study will use a randomized controlled trial design with two parallel arms to compare therapist-guided e-CBTi and trazodone’s treatment effects on sleep outcomes in patients with insomnia. The nature of the comparative effectiveness study will facilitate the investigation of the interventions’ effects under the circumstances that are comparable with clinical settings, which may provide important insights on the interventions’ impacts when integrated into routine clinical practices in the future. In addition, the SPIRIT guideline that outlines the standard evidence-based protocol items in intervention trials has been followed. Fig 1 outlines the schedule for enrollment, intervention, and assessments. This study is a single-site study, and it will run through the Queen’s University Online Psychotherapy Lab (QUOPL), Kingston, Canada. See Fig 2 for the participant flow chart. Ethical approval was received from the Queen’s University Health Science and Affiliated Teaching Hospitals Research Ethics Board (HSREB File #: 6032770). The study is registered with ClinicalTrials.gov under registration number NCT05125146.

### Sample size

A priori power analyses using the G*Power V3.1 software indicated that a sample size of 36 participants would provide adequate power to detect a medium (f = 0.25) group x time interaction effect using repeated measures analysis of variance (ANOVA) with four sequential measurements per subject per week when the correlation among the measures was 0.3 (α = 0.05). Given the likelihood of participant dropout or withdrawal, participants have been purposely over-sampled to obtain meaningful and statistically significant results regarding changes in their symptoms at the end of the study. Based on previous experience with e-CBTi in similar patient populations, a dropout of up to 25% is anticipated [38]. Considering that former studies of similar nature have suggested that N = 20, 30, 24 per study arm has produced statistically significant interaction effects [39–41]. A sample size of 30 participants in each arm or 60 participants in total, is planned to be recruited for this study to detect significant results with p = 0.05 after expected attrition.

### Participants

Patients (n = 60) diagnosed with insomnia will be recruited through referrals from health care professionals at Kingston Health Sciences Centre (Kingston, Canada), family physicians, and other health care providers or self-referrals in Kingston, Ontario, Canada. Patients who show interest in the study will then be advised to meet with a study research coordinator who will provide them with letters of information containing study details. If patients wish to participate, written and verbal consent is required for participation and will be collected by the research coordinator.

After consenting to participate in the study, a mini-international neuropsychiatric interview (MINI) based on the Diagnostic and Statistical Manual of Mental Disorders, Fifth Edition (DSM-5) will be completed by a psychiatrist on the research team to confirm diagnosis and eligibility [42]. Inclusion criteria include the following: at least 18 years of age at the start of the study, non-organic insomnia (insomnia not due to other psychiatric disorders, or medical conditions); the presence of insomnia symptoms for at least 3-month; difficulty initiating and/or maintaining sleep for ≥ 30 minutes; ability to speak and read English; consistent and reliable access to the internet; and not using or changing the dose of sleep medications within the six weeks before commencing the study [43]. Patients diagnosed with insomnia of all severities according to DSM-5 will be included in this study. Exclusion criteria include the presence of another untreated sleep disorder requiring other treatments such as sleep apnea or alcohol, or substance use disorder, ongoing CBT, and non-assessed or untreated somatic symptom disorder according to the DSM-5. Pregnant women or participants who may become pregnant will be excluded from participation owing to concerns about potential adverse fetal effects from trazodone.

### Blinding, randomization and treatment allocation

An independent researcher will conduct participant randomization using the MS Excel randomization function with a 1:1 ratio. This will determine whether participants are assigned to the e-CBTi or the pharmacotherapy group. Blinding of participants and therapists directly involved in patient care is not possible due to the nature of this study. However, all people involved in outcome assessments and data analysis will be blinded from the group assignment.

### Procedure

Upon completion of the initial assessment and treatment allocation, all eligible participants will be added to a secure online platform, OPTT. The OPTT system is designed by the lead author, and developed in collaboration with a team of physicians, and web developers with expertise in online CBT, neuroscience, and data. The user can operate the OPTT system on any electronic devices connected to Wi-Fi. All e-CBTi sessions and therapist-participant interactions during the study period will occur over this platform. The same set of therapists of the same orientation, training, shared environment, and supervision is used between groups to control for the potential covariates impacting the therapeutic relationship and minimize the potential of the therapist’s effects on patient outcomes. At baseline (week 0), all participants will complete a demographic survey, and clinically validated sleep questionnaires. Online questionnaires will also be completed weekly during the study period, and post-intervention (week 7–8). Participants assigned to the psychotherapy group will receive e-CBTi along with their treatment as usual (TAU) for seven weeks. TAU is defined as the standard psychiatric or pharmaceutical treatment for patients’ conditions with no restrictions apart from the use of the experimental treatment (trazodone and CBT). If TAU includes a medication that could affect sleep, the dosage must remain consistent 6 weeks prior to the study and during the study period. Participants assigned to the pharmacotherapy group will be prescribed trazodone in the first session in addition to TAU. Once post-test questionnaires are complete, participants allocated to the pharmacotherapy group will also have the opportunity to participate in the therapist-guided e-CBTi program after the study period.

### Pharmaceutical intervention

Participants allocated to the pharmacotherapy group will be prescribed trazodone as a regular treatment for the study duration. Participants will be initially instructed to meet with the psychiatrist on the research team through video sessions. The psychiatrist will provide information on trazodone usage, possible side effects, and drug interactions. All participants in the pharmacotherapy group will receive a starting dose of 50mg/day at their first session and are encouraged to contact the psychiatrist’s office if they experience any side effects or have concerns about the medication. The psychiatrist will get back to them via phone on the same day and discuss potential dosage adjustments according to the participants’ circumstances. Additionally, a psychiatrist will follow up with the participant mid-program (week 4) via video sessions. During these follow-up appointments, dosage may be adjusted according to drug effectiveness and possible adverse effects. The maximum dosage for trazodone is 100mg/day for this study. These low doses (50mg-100mg) enable trazodone to produce hypnotic effects without causing tolerance or daytime drowsiness [11]. Participants will continue to take their medication as directed and meet with the psychiatrist again at the end of the study. If participants decide to continue with trazodone or wish to discontinue medication, their requests will be communicated to their family physician for any further care.

### E-CBTi intervention

Participants allocated to the psychotherapy group will receive e-CBTi during the study period. The program content involves interactive and engaging weekly therapy sessions. Participants will receive personalized feedback from a trained therapist each week. All sessions and interactions will occur through a secure online platform, OPTT. Pre-designed therapy sessions will be assigned to the patients through the platform and can be accessed at any time throughout the week. Each session consists of approximately 30 slides that take approximately 45 minutes to complete. The e-CBTi program is based on the rationale that insomnia is associated with thoughts and behaviours that cause or worsen sleep problems [14]. The e-CBTi sessions will guide participants in developing constructive and balanced strategies to help manage their sleep. The sessions aim to adjust maladaptive thinking and behaviours so that participants can use skills and strategies to think about and adapt to the events happening to them. This approach enables individuals to, allowing them to adjust their behaviour and thoughts to ones that are more balanced and realistic. Each of the structured e-CBTi sessions will focus on the concepts listed in Table 1.

**Table 1:** Session overview.

Session	Learning Objective / Session Description
Session 1: What is Sleep?	Provides an overview of the course and introduces the concepts of sleep, CBT, and insomnia.
Session 2: Sleep Habits	Discusses various sleep habits’ impacts on sleep. Provides stimulus control instructions, such as activities that are permitted or disallowed when in bed, daytime napping and what to do if sleep is not attained at night. Provides sleep restriction instructions, including setting specific bedtime, maintaining the same wake time, and restricting time spent in bed awake.
Session 3: Sleep Hygiene	Provides sleep hygiene education and discusses behavioural health practices, such as caffeine, alcohol, exercise, diet, smoking, and environmental factors’ impacts on sleep. Provides an overview of relaxation techniques that support sleep.
Session 4: Bedtime Worries	Introduces negative thoughts’ roles in insomnia and strategies to manage disturbing and unhelpful thoughts through the constructive worries exercise.
Session 5: Negative Thoughts	Explains the role of automatic thoughts and how they influence feelings. Provides ways to identify unhelpful thinking errors related to sleep.
Session 6: Thought Examination	Discusses elements of the thought examination process, including identifying automatic negative thoughts about sleep, evidence that supports or does not support those thoughts, alternative views of the situation, and feelings associated with the situation.
Session 7: Review	Summarizes the main concepts of CBT and the tools and skills the patient should continue to practice beyond completion of the final session.

Each session will end with a homework assignment for patients to complete, including symptom logs, self-reflective questions, and specific structured exercises such as the thought examination worksheet. Participants will submit their homework directly to the therapist through the OPTT platform. Trained therapists will review participants’ homework, sleep diary, weekly insomnia ratings, and Fitbit data and provide personalized feedback within 48 hours through OPTT. Pre-designed homework feedback templates, including session contents summary and evidence-based information, are provided to therapists as a basic structure to help standardize care, ensure feedback quality, and promote efficiency. With a particular focus on patient-centredness and self-management, therapists then personalize the feedback template to address patient-specific challenges, such as unhelpful thought patterns related to insomnia and sleep habits. Once patients receive feedback, they can ask questions, and therapists can respond to those questions under the same thread. During each session, patients and therapists can also communicate through the chat box to discuss patients’ progress and address potential challenges experienced.

### Training

All therapists will be research assistants hired by the principal investigator. They will be undergraduate and master’s students at Queen’s University with relevant psychology backgrounds and clinical experiences related to psychotherapy. They will all have undergone weekly training in CBT, psychotherapy delivery, and additional training from a licenced psychotherapist on the research team for two months before interacting with participants. During training, therapists also practice completing feedback on practice homework templates. A lead therapist on the research team will then review them and provide feedback. Following the training, all therapists will be supervised by the lead psychiatrist, an expert in e-CBTi, to provide personalized feedback and guidance for patients during the e-CBTi program. Before submitting feedback to a participant, it is reviewed by the lead psychiatrist to check for adherence to treatment content and treatment fidelity.

### Outcome evaluation

All participants are expected to wear the Fitbit sleep tracking device during the study duration and complete the Insomnia Severity Index (ISI) questionnaire and sleep diary on the OPTT platform at baseline (week 0), weekly during the study, and post-treatment (week 7–8). A tutorial for using the sleep diary on the platform will be provided to all participants. An independent researcher will collect all quantitative and qualitative measures by extrapolating recorded information through the OPTT platform.

### Primary outcome

*Insomnia Severity Index (ISI)* The primary outcome measure will be ISI [44]. ISI is a valid, reliable, and sensitive global index of self-reported insomnia symptoms that contains seven questions rated from 0 to 4 on the severity of insomnia problems, sleep satisfaction, interference of insomnia with daytime functioning; noticeability of sleep problems by others; and distress about sleep difficulties. Psychometric parameters of ISI are adequate, sensitive to change, and have been validated for online use [45].

### Secondary outcomes

Several secondary outcomes, including time in bed (TIB), sleep efficiency (SE), sleep onset latency (SOL), wake time after sleep onset (WASO), number of nocturnal awakenings (NOW), total sleep time (TST), and sleep quality (SQ) will be derived from both sleep diaries and Fitbits to assess changes in sleep outcomes from pre-treatment to post-treatment.

*Sleep Diary* A sleep diary will be used to derive secondary sleep outcomes [46]. Sleep diary has been widely used for self-reported sleep parameter assessment, and it is a reliable tool for assessing adult sleep time and quality [47]. Participants are asked to complete a web-based sleep diary on the OPTT platform for at least four days each week within thirty minutes of waking up. Four nights is chosen as the minimum required per week because a study conducted by Short et al. showed adequate reliability of four nights of sleep diary entries for assessing bedtime, sleep duration, and SOL [48]. Participants will record their bedtime, time of falling asleep, nighttime awakenings, wake-up time, and time of getting out of bed. Subjective sleep quality is rated weekly on a 1–5 scale ranging from “very poor” to “very good.”

*Fitbit* The study will also observe the data obtained from a wearable tracker (Fitbit). Participants will be provided with a Fitbit wristband to use: Fitbit Charge 2. Fitbit contains heart rate sensors and motion detectors which facilitate sleep monitoring [49]. Fitbit’s accuracy and strength in evaluating sleep are comparable to polysomnography, actigraphy, and self-reported measures, while providing less resource-intensive sleep monitoring than these instruments, which supports the reliability of using Fitbit to monitor sleep in this study [50–53]. However, despite the similarity between Fitbit and research-grade devices such as actigraphy and polysomnography performances, caution should be taken in the interpretation of the use of consumer technology [50,53]. Participants are expected to always wear the Fitbit sleep tracking device during the study duration. Data will be collected using the Fitbit application that is synced with the device and then automatically uploaded to the secure OPTT web server. Upon completion of the study, the Fitbit will be returned to the lab, and Fitbit data collected via OPTT will be used for further analyses. The combination of the subjective sleep diary, ISI questionnaire and objective Fitbit measures will be beneficial for assessing patients’ sleep outcomes and treatment effectiveness in this study.

### Additional measures

Participants will also report their baseline demographics, including their age, sex, income, ethnicity, marital status, native language, employment status, current medication usage, living arrangements, education level and medical history. Finally, quantitative behavioural measures for both groups, including the number of logins, the number of homework and questionnaires completed, and the number of sessions completed will be collected to assess patients’ engagement and adherence with the treatment. Additionally, treatment adherence in the e-CBTi group will be further explored by assessing compliance to keeping a consistent sleep schedule, avoiding naps, eliminating consuming alcohol or nicotine 2–3 hours before going to bed, getting out of bed shortly after waking and completing thought examination practices based on results from the sleep diary, Fitbit and homework for each session [54].

### Ethical considerations and data management

Patient confidentiality and data privacy will be protected throughout the study, including assessment, treatment, and data analysis, in accordance with Queen’s University ethics guidelines. All study personnel will be trained in responsible and ethical research conduct and the handling of private and confidential information through the completion of the tri-council policy statement: ethical conduct for research involving humans (TCPS 2) research ethics course prior to their involvement in the study.

All participants are expected to provide electronic or written informed consent for data to be collected before participating in the study. To ensure the safe handling of data and protect confidentiality and privacy, all research participants will be provided with a unique and randomized participant login ID and password for OPTT. All data is stored through OPTT. Data management through OPTT is in accordance with the Health Insurance Portability and Accountability Act (HIPAA), the Personal Information Protection and Electronic Documents Act (PIPEDA), and the Service Organization Control-2 (SOC-2). Moreover, data collected from OPTT is sent to secured servers and databases hosted on Amazon Web Service Canada cloud infrastructure, managed by Med stack to ensure all Canadian provincial and federal privacy and security regulations are met. OPTT will not collect identifiable personal information or IP addresses for privacy reasons. OPTT will only collect anonymized metadata to improve its service quality and provide advanced analytics data to the research team. All encrypted backups will be kept on secure Queen’s University servers. OPTT automatically encrypts all data, and no employees have direct access to patient data. Only the direct care provider, independent biostatistician, and research assistants will be able to access anonymized study records to facilitate assessment, the welfare of participants, and data analysis. Any documents with identifiable information (e.g., consent forms and personal information) will be stored locally in a locked file cabinet or online in password-protected formats. They will be stored separately from the anonymized data and may only be accessed to be integrated into data interpretation for research purposes.

### Safety considerations

Studies conducted in similar clinical populations demonstrated that low doses (25mg-100mg/day) of trazodone for managing insomnia produced mild, including daytime sleepiness and mild headaches, to no adverse effects [11]. Additionally, previous studies in similar samples have also shown that e-CBTi can be safely carried out without significant risks of unwanted effects.

Participation in this study is voluntary. Participants may withdraw from this study at any time without providing a reason, and their data will remain confidential. Participants’ withdrawal will not affect their current or future medical care, and they may continue receiving standard care procedures.

### Statistical analyses

SPSS version 25 will be used to analyze the data (e.g., analyses of missing values, baseline characteristics, the effect of the intervention, compliance rates). For all analyses, the alpha level will be set to 0.05. Initially, all data will be examined for missing, nonsensical, and outlying variables. Missing data will be treated as missing and not imputed. Participants who did not complete all seven sessions of e-CBTi or trazodone are considered dropouts. Additionally, dropout rates between groups will be compared using chi-square tests, and demographic and dependent variables will be assessed and compared between completers and participants who withdraw prematurely to estimate dropout effects. Descriptive statistics will be used to describe the sample’s baseline demographics. Group differences in baseline demographics will be analyzed using independent sample t-tests for continuous variables (e.g., age, income). Chi-square test will be used to examine group differences in categorical variables (e.g., sex, ethnicity, native language, employment status, children, current medications, education level, medication usage, living arrangements, and primary diagnosis). Data from all participants will be included in the analyses. Treatment effectiveness will be assessed using two-way ANOVA for repeated measures and multilevel regression analysis with the main effects as the group (e-CBTi vs trazodone) and time (pre-treatment to post-treatment). The group x time interaction effects for the ISI scores, sleep diary measures, and Fitbit sleep estimates will be evaluated to compare the change from pre-treatment to post-treatment between and within the groups. Descriptive statistics will be used to describe the sample and patient engagement, including the number of logins, the number of sessions completed, and the number of homework or questionnaires submitted. An independent sample t-test will be used to identify group differences regarding these behavioural variables.

## Results

The study was reviewed for ethical compliance and ethics approval was received from the Queen’s University Health Science and Affiliated Teaching Hospitals Research Ethics Board in October 2021. Participant recruitment began in November 2021. Recruitment has been conducted through social media advertisements, physical advertisements, and physician referrals. To date, 18 participants have been recruited (e-CBTi: n = 11; trazodone: n = 7). Data collection is expected to conclude by December 2022, and data analyses are expected to be completed by January 2023 with analysis of variance (ANOVA) for continuous outcomes (e.g., ISI score, sleep diary, Fitbit), and chi-square tests for categorical outcomes (e.g., sex, ethnicity, employment status). Any modifications to the study will be submitted as amendments. The study results will be published in peer reviewed journals and a lay summary will be sent to interested participants.

## Discussion

The pharmaceutical drug (trazodone) and in-person CBTi are common therapeutic approaches to manage insomnia [5,6]. However, many individuals fail to adhere to pharmacotherapies due to stigma, drug-drug interactions, and unpleasant side effects [12]. Uptake of CBTi is also poor for several reasons, including high costs, long wait time, and inaccessibility to services [20]. Emerging evidence indicates that increasing the availability of CBTi may be critical to further improving the accessibility to quality treatment for insomnia. By designing treatment programs that deliver CBT through a secured online web-based format with asynchronous therapist guidance, accessibility and adherence to CBT are likely to be enhanced [22,30,35]. Currently, studies that examine asynchronous therapist-guided e-CBTi are limited. This study is an extension of the research team’s prior work on interactive CBT sessions and personalized feedback delivered through OPTT in managing other mental disorders [55–58]. The current protocol describes the rationale and design of a 2-arm RCT that aims to assess the comparative effectiveness of therapist-guided e-CBTi through OPTT relative to one of the commonly used sleep medications (trazodone) in insomnia patients.

Several strengths of the study design are worth noting. The e-CBTi program is designed to enhance patient engagement and adherence to their treatment. With asynchronous communications in conjunction with interactive CBTi contents, the e-CBTi program aims to strengthen the therapist-patient connection to better support patients in developing coping skills to tackle insomnia episodes in the long term. Furthermore, unlike previous studies that delivered asynchronous communications via email, patient communications occur asynchronously via a more secure and scalable online web-based platform, OPTT [33,34]. Finally, in contrast to previous studies that limited therapist-guided e-CBTi’s comparison to waitlist control or internet-based controls, this study directly compares it to commonly prescribed medications (trazodone) [33,34]. Another unique aspect of the study includes collecting objective sleep data via wearable sensors, Fitbit, in combination with subjective self-reported measures to assess treatment effects. One of the limitations of the study design is that participants can be recruited from the general population through self-referrals, which could have biased the level of motivation compared to patients whom physicians have referred. Therefore, future research could explore the effects of motivation and self-selection for treatment on the effectiveness of trazodone and therapist-guided e-CBTi in this clinical population. Furthermore, another limitation of the design is that there is no follow-up time point in the current study. Considering the long-term benefits of the e-CBTi, building upon findings from this study, future studies can include follow-ups to explore the long-term effects of e-CBT compared to pharmaceutical intervention in this population.

Outcomes from this study will provide evidence for a novel, accessible, and scalable treatment model for adults with insomnia. From a clinical perspective, study results could help clinicians who support insomnia patients identify efficacious treatments for this population. Findings may also inform future research, including the dissemination and cost-effectiveness of therapist-guided e-CBTi in clinical settings. Finally, a more accessible and affordable mode of delivering CBT could inform public health policy-makers to integrate such interventions in clinical and community settings to enhance patient care.

## Supporting information

S1 Table SPIRIT 2013 checklist: Recommended items to address in a clinical trial protocol and related document. (PDF)

S1 File Study protocol approved by the ethical committee. (PDF)

## Abbreviations

CBT: cognitive behavioural therapy
CBTi: cognitive behavioural therapy for insomnia
e-CBT: electronically-delivered cognitive behavioural therapy
e-CBTi: electronically-delivered cognitive behavioural therapy for insomnia
ISI: Insomnia Severity Index
NOW: number of nocturnal awakenings
NWAK: nocturnal awakenings
OPTT: Online Psychotherapy Tool
SE: sleep efficiency
SOL: sleep onset latency
SQ: sleep quality
TAU: treatment as usual
TIB: time in bed
TST: total sleep time
WASO: wake after sleep onset
