# Supplementary material for: Investigating the effectiveness of electronically delivered cognitive behavioural therapy (e-CBTi) compared to pharmaceutical interventions in treating insomnia: Protocol for a randomized controlled trial
Source: PLoS One. 2023 May 16;18(5):e0285757. doi: 10.1371/journal.pone.0285757 (PMC10187924; doi:10.1371/journal.pone.0285757)
Supplement: S1 File — (PDF) [file pone.0285757.s002.pdf]

Investigating the effectiveness of electronically delivered cognitive behavioural therapy (e-CBTi) compared to pharmaceutical interventions in the treatment of insomnia

Authors/Contributions

Principle Investigator: Dr. Nazanin Alavi MD FRCPC[a,b]

Co-Principle Investigator: Dr. Megan Yang MD[a]

Collaborator: Dr. Mohsen Omrani MD PhD [a,b,c]

Co-Investigators: Callum Stephenson BScH[a,b], Jasleen Jagayat BScH [a,b], Dr. Niloofar Nikjoo MD [a], Yiran Zhu[d]

a. Department of Psychiatry, Queen’s University, Kingston, ON, Canada
b. Centre for Neuroscience Studies, Queen’s University, Kingston, ON, Canada
c. OPTT Inc., Toronto, ON, Canada
d. Faculty of Health Sciences, Queen’s University, Kingston, ON, Canada

Registration The registration number will be provided when it is available and obtained.

Corresponding Author

Nazanin Alavi MD FRCPC
Department of Psychiatry, Queen’s University
166 Brock Street, Kingston, ON, Canada K7L 5G2
Email: nazanin.alavi@queensu.ca
Phone: 1-613-544-3310
Fax: 1-613 544-9666

Abstract

Insomnia is defined as the inability to fall asleep or stay asleep at night and it is one of the most prevalent sleep disorders that can have deleterious impacts on health and this population's quality of life. Currently, both pharmaceutical interventions (trazodone) and cognitive behavioral therapy (CBTi) are widely used to treat patients with insomnia (Saddichha,2010). Although CBTi has been efficacious in many patients, multitude of barriers for receiving treatment such as its limited availability of therapists, high costs and long wait times challenge its ability in sufficiently meeting the population's health needs and demands (Okajima et al., 2010; Taylor &Pruiksma, 2014; Edinger & Means, 2005; Kaltenthaler & Cavanagh, 2010). To improve the delivery of CBT, electronically delivered CBTi (e-CBTi) has been developed as an accessible and effective alternative intervention for improving sleep outcomes in patients with insomnia. While evidence suggest that e-CBTi is effective when compared to placebos/waitlist control, evidence comparing guided e-CBTi to pharmaceutical interventions is still insufficient and needs further exploration (Ye et al.,2016; Seyffert et al.,2016).

Through this proposal, we aim to implement a guided electronically delivered CBTi to address insomnia in the study population and compare its treatment effectiveness to trazodone. All eligible participants (n-60) will be randomly assigned to two groups: Treatment as usual (TAU) + medication (trazodone), and TAU+ e-CBTi for 7 weeks.7 sessions of sleep modules will be used, with the Online Psychotherapy Tool (OPTT), a secure cloud-based platform for the online delivery of psychotherapy developed by the PI- Dr. Alavi. We will evaluate how patients' mental status and symptoms of insomnia change through this intervention by assessing Insomnia Severity Index (ISI), standard sleep questionnaires, and other behavioural variables.

Introduction

Background and Rationale

Insomnia is a common sleep disorder that can have many serious health impacts on patients (Roth,2007). Worldwide, epidemiologic studies have shown that insomnia is prevalent in 10% - 30% of the general population, some as high as 50-60%, and it is especially common in older adults, females, and people with other medical and mental illnesses (Bhaskar, Hemavathy, & Prasad, 2016). Insomnia refers to difficulty with sleep initiation, consolidation, duration, quality, and maintenance that significantly impairs patients' quality and opportunity for sleep (Roth,2007). As a result, this can have deleterious impacts on health, leading to increased risks for mental illnesses, accidents, poor physical health, and shortened life expectancy that can place a heavy disease burden on this population (Colten & Altevogt, 2006).

Pharmaceutical interventions and cognitive behavioural therapy (CBTi) are the most supported therapeutic approaches for managing insomnia (Saddichha,2010). Pharmacotherapeutic agents such as benzodiazepines and zopiclone have shown sufficient evidence of their efficacy and safety for short term alleviation of insomnia (Saddichha,2010). While trazodone is approved for the treatment of depression, it is also one of the most prescribed sleep aids for the management of insomnia (Jaffer et al.,2017; Lie et al.,2015). A systematic review that screened 45 studies has provided adequate evidence supporting low dose use of trazodone's safety and efficacy in improving sleep outcomes by decreasing sleep latency and increasing sleep duration in patients, and it has been validated as an efficacious treatment for insomnia (Jaffer et al.,2017).

In addition to insomnia medications, non-pharmaceutical cognitive behavioural therapy is also effective in improving sleep outcomes in adults with insomnia. It has been recommended as the first line of treatment for people with insomnia (Baglioni et al., 2019). Several meta-analyses and systematic reviews have shown CBTi to be effective in improving insomnia severity, subjective sleep quality, total sleep time, and other sleep-related parameters post-treatment when compared to waitlist control, placebo or pharmaceutical interventions (Okajima et al., 2010; Taylor &Pruiksma, 2014; Edinger & Means, 2005). While CBTi has been efficacious in many patients, multitude of barriers such as its limited availability of therapists, high costs and long wait times

challenge its ability in meeting the population's health needs and demands (Kaltenthaler & Cavanagh, 2010).

As a means of enhancing the availability of CBTi, electronically delivered CBT (e-CBT) emerges as an effective alternative psychological approach in managing mental illnesses, such as insomnia. This method deliver psychotherapy based on CBT principles through electronic devices. There has been increased research focused on the efficacy of e-CBT as it addresses many challenges faced by traditional in-person CBT. Based on the available evidence for the efficacy of e-CBT for different mental health disorders across meta-analysis and systematic reviews, e-CBT has shown great efficacy in the treatment and management of various psychiatric disorders such as depression, anxiety, and substance use disorders (Kumar et al., 2017; Zhou et al.,2016; Etzelmueller et al.,2020). Moreover, multiple meta-analysis reviews also suggest that e-CBT not only was effective in improving mental illness symptoms but also provided more accessible, cost-effective, and flexible mental health treatment for patients (Musiat et al.,2014; Andersson, Goldstone&Tarrier,2014). E-CBTi has been suggested as an effective way of improving sleep outcomes in patients with insomnia (Ye et al.,2016; Espie et al., 2012; Hagatun et al.,2017; Ritterband et al.,2017). Most e-CBTi provides online treatment modules on sycho-education, sleep hygiene, sleep restriction, and different cognitive exercises (Vincent & Lewycky,2009). Past literature provides evidence in support of the efficacy of online CBTi in insomnia in which sleep outcome is significantly improved following the treatment when compared to waitlist controls (Ye et al.,2016). Therefore, e-CBTi has gained popularity and has become a valuable method for treating insomnia.

While trazodone and e-CBTi have been effective in the management of insomnia, few investigations have directly compared their relative effectiveness in treating insomnia. Among the clinical trials conducted, e-CBTi treatments efficacy has been mostly compared to wait-list control/placebo, and rarely to medications (Ye et al.,2016). Investigations have also mainly focused on unguided and self-administered electronically delivered treatment for insomnia while recent evidence has suggested that guided e-CBTi treatment with a greater level of care provider engagement might further enhance the quality of care delivered. To address this research knowledge gap, the primary objective of this study is to investigate the effectiveness of guided electronically delivered CBT for insomnia (e-CBTi) compared to pharmaceutical intervention

(trazodone) in the management of insomnia. While our primary outcome of interest is Insomnia Severity Index (ISI), secondary outcomes are total sleep time, sleep onset latency, number of nocturnal awakenings, and sleep quality. These clinical findings may provide direct implications for the development of clinical guidelines for managing insomnia conditions in the future and encourage appropriate use of e-CBTi as a novel model for insomnia treatment to enhance patients' health outcomes.

Objective

This study aims to investigate the treatment efficacy of electronically delivered CBT (e-CBTi) compared to pharmacotherapy- trazodone in the management of insomnia. We hypothesize that electronically delivered CBT for insomnia (e-CBTi) will improve health outcomes of participants in several sleep-related parameters when compared to trazodone:

1. Patients will report lower Insomnia Severity Index (ISI)
2. Patients will report longer total sleep time (TST)
3. Patients will report shorter sleep onset latency (SOL)
4. Patients will report less number of nocturnal awakenings (NWAK)
5. Patients will report improved sleep quality

Methods: Participants, Interventions, outcomes

Population/recruitment/ Inclusion Exclusion Criteria

n=60 diagnosed with insomnia will be recruited to the study through referrals from health care professionals at Kingston Health Sciences Centre (KHSC), family physicians, and other health care providers or self-referrals. After consenting to participate in the study, an initial psychiatric assessment will be completed by one of the psychiatrists on the study to confirm the diagnosis and eligibility to take part in the study. Inclusion criteria: age of 18 years old or older, non-organic insomnia (F51.0) (World Health Organization 1995); difficulty initiating [sleep onset

latency (SOL)] and/or maintaining sleep [wake after sleep onset (WASO)] for $\geq$ 30 min and/or use of sleep-promoting medication (SPM) at least three nights per week for at least 3 months (Buysse et al. 2006); speak and read English, and consistent and reliable access to the internet. Exclusion criteria include the presence of another untreated sleep disorder such as sleep apnea, alcohol or substance use disorder, ongoing cognitive-behavioral psychotherapy, non-assessed or untreated clinically significant somatic or mental symptoms or illnesses, or other sleep disorders that could explain current insomnia symptoms or interfere with or be worsened by CBT-i. Alcohol and substance use will be evaluated during the initial assessment by a psychiatrist on the research team. If the participant meets the criteria for a substance use disorder, they will be excluded from the study and directed to the proper resources (i.e., support groups). Pregnant women are excluded from participation owing to concerns about potential adverse fetal effects of treatment as there are no adequate and well-controlled studies on trazodone in pregnant women for it to be deemed completely safe for use during pregnancy. During the initial assessment before enrollment, participants will be explained thoroughly potential risks and benefits of trazodone in pregnancy and will be asked if they are or think they may be pregnant. Pregnant participants or participants who think that they may be pregnant will be excluded from participation in the trial. This pregnancy testing plan can minimize potential fetal risks and protect women from potential harm while minimizing participants' burdens and maximizing the benefits of participation in the clinical trial for females of reproductive potentials. Additionally, if a participant thinks that they may be pregnant or becomes pregnant during the duration of the study, they will be instructed to meet with the psychiatrist and be removed from eligibility, terminating study participation. Participants will be advised to speak with the doctor and the doctor will help the participant to decide the treatment option to withdraw trazodone that is best for her and her baby. To withdraw trazodone, the dosage of trazodone should be tapered off gradually under a doctor's advice.

Procedure

Upon completion of the initial assessment which determines eligibility, all eligible participants will then be randomly assigned to two groups using the online randomization (https://www.graphpad.com/quickcalcs/index.cfm). Participants assigned to group A will be prescribed trazodone in addition to their treatment as usual (TAU), and participants assigned to

group B will receive e-CBTi along with TAU for 7 weeks. Patients allocated to the e-CBTi experimental group will receive a 7-week online program in addition to treatment as usual. The content of this program will involve interactive and engaging therapy modules, for which participants will receive individualized feedback from a therapist each week. All online sessions and interactions will occur through a secure online platform (OPTT). Through the platform, the pre-designed therapy modules are assigned to the patients and are accessible to them at any time through the week. Each module consists of approximately 30 slides, which take an average of 45 minutes to complete. Every week participants will submit homework, which is then directly submitted to the therapist that will provide personalized feedback to each patient. The pharmacotherapy group will be prescribed trazodone in addition to their TAU for 7 weeks, which has been scientifically proven to effectively treat insomnia. They will also have the opportunity to take part in the e-CBTi after the 7 weeks study period. Participants in the pharmacotherapy group will be asked to complete the same questionnaire as the e-CBTi group. All sleep diary and ISI information is collected via OPTT regardless of study arms.

Pharmaceutical Intervention

Trazodone is the treatment of choice for insomnia and participants allocated to the pharmaceutical intervention group will be prescribed trazodone as a regular treatment for the duration of the study. Participants on trazodone will also visit the psychiatrist every month to ensure that the wellbeing of the participants is protected, the quality of the data is maintained, and the conduct of the trial is in compliance with the approved protocol, and other regulatory requirements. Trazodone is the routine practice for insomnia and trazodone is covered for most patients, however, if the patient is not covered for trazodone, the research lab will provide gift cards available in the lab to accommodate the $50/month for the cost of trazodone.

e-CBTi intervention: Online Module Content

The e-CBTi modules will involve guiding participants to develop constructive and balanced strategies that would help assist participants to handle sleep problems that are affecting their life. The Online CBTi is based on a simple but powerful idea that insomnia is caused by thoughts and behaviors that can be changed, thus the module aims to adjust the negative thinking so that they can think about and adapt to the things that are happening to them; which allows participants to

adjust the way they behave and think about their problems in a way that is not as negative and instead in a way that is potentially more realistic and productive. The module content is designed to help patients with insomnia to deal with inaccurate thoughts about sleep and negative sleep behaviours effectively, change participants' lifestyle practices that negatively affect their sleep, and improve relaxation skills to improve healthy sleep patterns. More specifically, we focus on addressing and exploring the concept of sleep, sleep habits, sleep hygiene, bedtime worries, negative thoughts, and thoughts examination during our module to assist participants in working on thought processes, challenging irrational thoughts, and replacing them with balanced alternative thinking related to sleep.

Training

All therapists are research assistants hired by the principal investigator. They all undergo training in psychotherapy and additional training from a psychiatrist on the research team before any interaction with participants. During this training, therapists' complete feedback on practice homework templates which are reviewed by a psychiatrist on the research team to ensure adequate quality of work. All therapists are supervised by the lead psychiatrist who is an expert in the area of electronically delivered psychotherapy modalities. Before any submission of feedback to a participant, it is reviewed by the lead psychiatrist.

Outcomes

The primary outcome measured in this study is the insomnia severity index (ISI) (Bastien et al.,2001). ISI is a valid, reliable, and sensitive global index of self-reported insomnia symptoms in insomnia treatment in which it contains 7 questions rated from 0 to 4 on the severity of initial, middle, and late insomnia problems; sleep satisfaction; interference of insomnia with daytime functioning; noticeability of sleep problems by others; and distress about sleep difficulties. Psychometric parameters of ISI are adequate, sensitive to change, and it has also been validated for online use (Morin et al.,2011)

Secondary outcomes will be measured using a sleep diary, which is a stable tool used in insomnia assessment. Participants are encouraged to complete it weekly 30 minutes after waking up and participants will record their bedtime, time of falling asleep, nighttime awakenings, time

of waking up, and time of getting out of bed. Subjective sleep quality is rated weekly on a 1-to-5-point scale from very poor to very good. From these data related to sleep, sleep efficiency, sleep onset latency, total sleep time and sleep quality can be calculated and estimated to assess any improvements in the sleep outcomes from receiving the online CBTi treatment.

Finally, intervention use, adherence and other behavioural data regarding patients' interaction and engagement with their therapy (e.g. number of logins per day, amount of time spent on each session) will be collected directly through the platform to provide further information. We expect the level of patient engagement with their care might have predictive power in therapy outcomes.

We anticipated quantitative outcomes such as improvement in the psychometric ISI score, questionnaire scores from the sleep diary, and high frequency of logins during the study period. Our anticipated qualitative outcome is positive feedback from the online CBTi participants and healthcare providers.

Methods: Data collection, management, analysis

Data collection and Data Management

Participants' baseline characteristics and personal information such as email, phone, and name will be collected through initial assessments at the beginning of the study period. Furthermore, personal information such as gender/sex, age, race, religion, and sexuality will be stored locally in a locked file cabinet and may only be accessed to be integrated in data interpretation for research purposes and permission to access the above information will be obtained through consent forms. Study records such as primary and secondary sleep outcomes, behavioural variables and qualitative feedback will be collected anonymously through the secure OPTT platform for the purpose of data analysis and for answering the research questions. All participant information will be stored locally in a locked filing cabinet in the principal investigator's office for 5 years after the study completion date, and through the use of password-protected files, and encryption when sending information through Microsoft Teams. A data collection form can be found in the attachment.

Participation in this study is voluntary. Participants may withdraw from this study at any time without providing a reason and the withdrawal will not affect their current or future medical care. If participants wish to withdraw and withdraw their study data, they may email opt4.ecbt@queensu.ca expressing this and their information and data will be deleted from the system by the researcher from analysis up to the time of study conclusion for publication.

Statistical Analysis Plan

Initially, all data will be examined for missing, nonsensical and outlying variables. Missing data will be treated as missing and not imputed (i.e. will be analyzed on a per-protocol basis). Given the likelihood of participant drop-out or withdrawal, we have purposely over-sampled our study and control groups in order to obtain meaningful and statistically significant results at the end of the study. Based on previous experience with CBT and e-CBT in similar patient populations we anticipate up to 30% drop-out by the end of the treatment. However, the remaining individuals will be able to provide statistically significant information regarding changes in their symptoms. Given that several outcomes will be used, it is difficult to calculate a single sample size, or provide a specific power calculation. However, if we use the ISI as an example, as it is common to all participants, a 30% change is considered clinically significant. Therefore – a sample size of 30 participants in each arm of the study would be sufficient for detecting significant results with $p=0.05$, and a power of 0.95.

Baseline and demographic data from individuals who drop out will be compared to those who finish to identify any fundamental differences between completers and non-completers. Linear regression models will be used to compare treatment and control outcomes while controlling for demographic variables such as age and gender. Dr. Yang will perform independent statistical analysis to confirm outcomes generated by the lab.

Impact

The efficacy of online CBT in managing other disorders, such as mood and anxiety, using our interactive modules delivered through OPTT has been demonstrated in our previous studies (Alavi et al. 2016; Alavi and Hirji 2020; Alavi and Omrani 2018; Sapru et al. 2016). The outcomes of these trials completed at Queen’s University are published in several professional

peer reviewed journals and have been presented at national and international conferences. Based on our previous findings that showed the efficacy of online psychotherapy, we believe that CBTi delivered through OPTT will also be effective in improving the mental health of patients with insomnia. It is an efficient and accessible method of treatment delivery as one care provider can deliver care to 3-4 patients at the same time through the delivery of pre-designed asynchronous sessions. Providing more service to the patient with a lower cost, shorter wait time and greater coverage, increasing the capacity, performance and efficiency of our healthcare system. Furthermore, care provided on this platform can also be tailored to the patient's individual needs, values and preferences to facilitate respectful and responsive patient-centered care.

Findings from this study can provide important evidence of a potentially new method to govern clinical policy, and may provide family medicine clinics, specialists, and insurance companies with a new mental health resource to better improve patients' health.

Methods: Monitoring

Research participants will be asked to systematically observe and record their feelings, emotions, behaviours through the OPTT platform. Research team members will routinely review the self monitored information and track client progress and to also monitor the safety of research participants. Dr.Yang (co-PI) will also be involved in all parts of the protocol and proposal. Participants on trazodone will also visit the psychiatrist every month. At midpoint of the intervention period, an interim analysis for safety will be conducted by a group of experts on the research team to analyze if there's a huge disparity in safety of the interventions. Based on outcomes of the interim analysis, necessary adaptations will be made to the trial by the PI and co-PI to ensure participants' safety during the study.

Ethics and Dissemination

This is a part of a research study through Queen's University and this protocol will be reviewed and approved for ethical compliance by the Queen's University Health Sciences and Affiliated Teaching Hospitals Research Ethics Board. The protocol, specific informed consent forms, letter

of information, recruitment materials and other requested documents and modifications will also be reviewed and approved by the Queen's University Health Sciences and Affiliated Teaching Hospitals Research Ethics Board.

Consent

Research team will obtain consent from patients willing to participate in the trial. Information sheets and consent forms are provided for all patients involved in the trial.

Confidentiality

All information obtained during this study is strictly confidential and participants' identity will be always protected unless required by law. Each participant will have a file through the web-based platform OPTT. This program is secure, and password protected. Messages and homework that participants submit via OPTT can only be accessed by Dr. Alavi and the facilitators directly involved in the care. The data from this research will be saved without any identifying information. Participants will be identified by an identification number in the data from this study. Data will be stored in password-protected encrypted files and will be available only to Dr. Alavi and the facilitators involved in participants' treatment. Participants will not be identified in any publication or reports. An encrypted, password-protected, participant master list will have participants' name and contact information for the purposes of reaching out to participants during the study. Any study data that is collected will only be identifiable with a participant ID number that will be randomly assigned to the participant, protecting their identity.

The only individuals who will have access to the study data during collection, use, analysis, dissemination, retention, and/or disposal are the members of the study team (investigators, statistician, transcriber, etc.). To mitigate potential conflicts of interest, Dr. Mohsen Omrani will not have access to data, recruitment, or analysis. The Queen's University Health Sciences and Affiliated Teaching Hospitals Research Ethics Board (HSREB) may require access to study-related records to monitor the ethical conduct of the research.

All encrypted files will be stored on a secure Queen's University server. Once printed, these forms will be stored in a locked filing cabinet in the principal investigator's office for 5 years after the study

completion date. Participant identity will be protected for knowledge dissemination and publication of results. All information obtained during this study is strictly confidential and participants' identity will be always protected unless required by law (child abuse/neglect, elder abuse, etc.).

Conflicts of Interest

Dr. Nazanin Alavi (PI) has co-founded the care delivery platform in use (i.e. OPTT) and has ownership stakes in OPTT Inc. To mitigate potential conflicts, Dr. Alavi will not be involved in consenting the patients or sending referrals to the study. Moreover, Dr. Yang (the co-PI) will oversee the study along with Dr. Alavi to mitigate this potential conflict. All the raw data is available to the co-PI Dr.Yang, who would perform independent statistical analysis to confirm outcome generated by the lab. All the protocols will be discussed with Dr. Yang, who will oversee the study and will meticulously follow the protocol to avoid any COI affecting the procedures or the outcomes.

Dr. Mohsen Omrani (collaborator) is the CEO of OPTT and has ownership stakes in OPTT Inc. As a physician and an innovator, he will be the technical liaison to translate the extensive clinical expertise of the team to a user-friendly product, easily accessible to patients. Dr. Nazanin Alavi (PI) has co-founded the care delivery platform in use (i.e. OPTT) and has ownership stakes in OPTT Inc. To mitigate potential conflicts of interest, Dr. Mohsen Omrani will not have access to data, recruitment, or analysis.

References

Alavi N, Hirji A. The Efficacy of PowerPoint-based CBT Delivered Through Email: Breaking the Barriers to Treatment for Generalized Anxiety Disorder. J Psychiatr Pract 26: 89– 100, 2020.

Alavi N, Hirji A, Sutton C, Naeem F. Online CBT Is Effective in Overcoming Cultural and Language Barriers in Patients With Depression. J Psychiatr Pract 22: 2–8, 2016.

Alavi N, Omrani M. Online Cognitive Behavioral Therapy: An e-Mental Health Approach to Depression and Anxiety. Springer, 2018.

Andersson, G., & Titov, N. (2014). Advantages and limitations of Internet-based interventions for common mental disorders. *World psychiatry : official journal of the World Psychiatric Association (WPA)*, *13*(1), 4–11. https://doi.org/10.1002/wps.20083

Baglioni, C., Altena, E., Bjorvatn, B., Blom, K., Bothelius, K., Devoto, A., . . . Riemann, D. (2019). The European Academy for Cognitive Behavioural Therapy for Insomnia: An initiative of the European Insomnia Network to promote implementation and dissemination of treatment. *Journal of Sleep Research*, *29*(2). doi:10.1111/jsr.12967

Bastien, C.,Vallieres,A., Morin,M. (2001). Validation of the Insomnia Severity Index as an outcome measure for insomnia research. *Sleep Medicine*, *2*(4), 297-307. doi:10.1016/s1389-9457(00)00065-4

Bhaskar, S., Hemavathy, D., & Prasad, S. (2016). Prevalence of chronic insomnia in adult patients and its correlation with medical comorbidities. *Journal of family medicine and primary care*, *5*(4), 780–784. https://doi.org/10.4103/2249-4863.201153

Colten, H. R., & Altevogt, B. M. (2006). *Sleep disorders and sleep deprivation: An unmet public health problem*. Washington, DC: Institute of Medicine.

Edinger, J. D., & Means, M. K. (2005). Cognitive–behavioral therapy for primary insomnia. *Clinical Psychology Review*, *25*(5), 539-558. doi:10.1016/j.cpr.2005.04.003

Espie, C. A., Kyle, S. D., Williams, C., Ong, J. C., Douglas, N. J., Hames, P., & Brown, J. S. (2012). A Randomized, Placebo-Controlled Trial of Online Cognitive Behavioral Therapy for Chronic Insomnia Disorder Delivered via an Automated Media-Rich Web Application. *Sleep, 35*(6), 769-781. doi:10.5665/sleep.1872

Etzelmueller, A., Vis, C., Karyotaki, E., Baumeister, H., Titov, N., Berking, M., . . . Ebert, D. D. (2020). Effects of Internet-Based Cognitive Behavioral Therapy in Routine Care for Adults in Treatment for Depression and Anxiety: Systematic Review and Meta-Analysis. *Journal of Medical Internet Research, 22*(8). doi:10.2196/18100

Hagatun, S., Vedaa, Ø, Nordgreen, T., Smith, O. R., Pallesen, S., Havik, O. E., . . . Sivertsen, B. (2017). The Short-Term Efficacy of an Unguided Internet-Based Cognitive-Behavioral Therapy for Insomnia: A Randomized Controlled Trial With a Six-Month Nonrandomized Follow-Up. *Behavioral Sleep Medicine, 17*(2), 137-155. doi:10.1080/15402002.2017.1301941

Jaffer, K. Y., Chang, T., Vanle, B., Dang, J., Steiner, A. J., Loera, N., Abdelmesseh, M., Danovitch, I., & Ishak, W. W. (2017). Trazodone for Insomnia: A Systematic Review. *Innovations in clinical neuroscience, 14*(7-8), 24–34.

Kaldo, V., Jernelöv, S., Blom, K., Ljótsson, B., Brodin, M., Jörgensen, M., . . . Lindefors, N. (2015). Guided internet cognitive behavioral therapy for insomnia compared to a control treatment – A randomized trial. *Behaviour Research and Therapy, 71*, 90-100. doi:10.1016/j.brat.2015.06.001

Kaltenthaler, E., & Cavanagh, K. (2010). Computerised cognitive behavioural therapy and its uses. *Progress in Neurology and Psychiatry, 14*(3), 22-29. doi:10.1002/pnp.163

Kumar, V., Sattar, Y., Bseiso, A., Khan, S., & Rutkofsky, I. H. (2017). The Effectiveness of Internet-Based Cognitive Behavioral Therapy in Treatment of Psychiatric Disorders. *Cureus, 9*(8), e1626. https://doi.org/10.7759/cureus.1626

Lie, J. D., Tu, K. N., Shen, D. D., & Wong, B. M. (2015). Pharmacological Treatment of Insomnia. *P & T : a peer-reviewed journal for formulary management, 40*(11), 759–771.

Morin, C. M., Belleville, G., Bélanger, L., & Ivers, H. (2011). The Insomnia Severity Index: psychometric indicators to detect insomnia cases and evaluate treatment response. *Sleep, 34*(5), 601–608. https://doi.org/10.1093/sleep/34.5.601

Musiat, P., Goldstone, P., & Tarrier, N. (2014). Understanding the acceptability of e-mental health - attitudes and expectations towards computerised self-help treatments for mental health problems. *BMC Psychiatry, 14*(1). doi:10.1186/1471-244x-14-109

Okajima, I., Komada, Y., & Inoue, Y. (2010). A meta-analysis on the treatment effectiveness of cognitive behavioral therapy for primary insomnia. *Sleep and Biological Rhythms, 9*(1), 24-34. doi:10.1111/j.1479-8425.2010.00481.x

Ritterband, L. M., Thorndike, F. P., Ingersoll, K. S., Lord, H. R., Gonder-Frederick, L., Frederick, C., . . . Morin, C. M. (2017). Effect of a Web-Based Cognitive Behavior Therapy for Insomnia Intervention With 1-Year Follow-up. *JAMA Psychiatry, 74*(1), 68. doi:10.1001/jamapsychiatry.2016.3249

Roth T. (2007). Insomnia: definition, prevalence, etiology, and consequences. *Journal of clinical sleep medicine : JCSM : official publication of the American Academy of Sleep Medicine*, *3*(5 Suppl), S7–S10.

Saddichha S. (2010). Diagnosis and treatment of chronic insomnia. *Annals of Indian Academy of Neurology*, *13*(2), 94–102. https://doi.org/10.4103/0972-2327.64628

Sapru I, Khalid-Khan S, Choi E, Alavi N, Patel A, Sutton C, Odejayi G, Calancie OG. Effectiveness of online versus live multi-family psychoeducation group therapy for children and adolescents with mood or anxiety disorders: a pilot study. Int J Adolesc Med Health , 2016. doi:10.1515/ijamh-2016-0069.

Seyffert, M., Lagisetty, P., Landgraf, J., Chopra, V., Pfeiffer, P. N., Conte, M. L., & Rogers, M. A. (2016). Internet-delivered cognitive behavioral therapy to treat insomnia: A systematic review and meta-analysis. *PLOS ONE, 11*(2). https://doi.org/10.1371/journal.pone.0149139

Taylor, D. J., & Pruiksma, K. E. (2014). Cognitive and behavioural therapy for insomnia (CBT-I) in psychiatric populations: A systematic review. *International Review of Psychiatry, 26*(2), 205-213. doi:10.3109/09540261.2014.902808

Vincent, N., & Lewycky, S. (2009). Logging on for better sleep: RCT of the effectiveness of online treatment for insomnia. *Sleep*, *32*(6), 807–815. https://doi.org/10.1093/sleep/32.6.807

Ye, Y., Chen, N., Chen, J., Liu, J., Lin, L., Liu, Y., . . . Jiang, X. (2016). Internet-based cognitive–behavioural therapy for insomnia (ICBT-i): A meta-analysis of randomised controlled trials. *BMJ Open, 6*(11). doi:10.1136/bmjopen-2015-010707

Zhou, T., Li, X., Pei, Y., Gao, J., & Kong, J. (2016). Internet-based cognitive behavioural therapy for subthreshold depression: a systematic review and meta-analysis. *BMC psychiatry*, *16*(1), 356. https://doi.org/10.1186/s12888-016-1061-9
